# Clinical significance of posterior talofibular ligament injury in chronic lateral ankle instability

**DOI:** 10.1007/s00402-024-05598-7

**Published:** 2024-09-30

**Authors:** Tomoyuki Nakasa, Yasunari Ikuta, Shingo Kawabata, Satoru Sakurai, Dan Moriwaki, Saori Ishibashi, Asyumaredha Asril Silan, Nobuo Adachi

**Affiliations:** 1 Department of Artificial Joints and Biomaterials, Graduate School of Biomedical and Health Sciences, Hiroshima University, Hiroshima, Japan; 2 Department of Orthopaedic Surgery, Graduate School of Biomedical and Health Sciences, Hiroshima University, Hiroshima, Japan; 3 Orthopaedic and Traumatology Division, Muhammad Djamil Central Hospital, Padang, Indonesia

**Keywords:** Chronic lateral ankle instability, Posterior talofibular ligament, Magnetic resonance imaging, Anterior talofibular ligament, Calcaneofibular ligament

## Abstract

**Purpose:**

Although arthroscopic repair of the anterior talofibular ligament (ATFL) is widely performed, the effect of posterior talofibular ligament (PTFL) injury on clinical outcomes remains unclear. This study aimed to evaluate the magnetic resonance imaging (MRI) findings of the PTFL in chronic lateral ankle instability (CLAI) and determine whether the presence or absence of PTFL injury affected the postoperative outcomes of arthroscopic ATFL repair.

**Materials and methods:**

Forty ankles of 35 patients who underwent arthroscopic repair for CLAI were included in this study as the experimental group, together with 25 ankles of 24 patients without CLAI as the control group. The PTFL thickness (PTFLT) and PTFL cross-sectional area (PTFLCSA) were measured using MRI and compared between the control and CLAI groups. The clinical outcomes of arthroscopic repair were compared between ankles with and without PTFL injuries.

**Results:**

The mean PTFLT and PTFLCSA values were significantly higher in the CLAI group than in the control group. The PTFLT and PTFLCSA in the PTFL injury group were significantly larger than those in the non-injury group in the CLAI group. Postoperatively, there were no significant differences in clinical scores and talar tilt angles on stress radiographs between ankles with and without PTFL injury; however, instability recurrence was frequently observed in ankles with PTFL injury (32.1%) compared to the ankles without PTFL injury (16.7%). Poor-quality ATFL remnant, ATFL inferior fascicle, and calcaneofibular ligament injuries were frequently observed in ankles with PTFL injuries.

**Conclusions:**

Our findings indicate that PTFL injury is highly associated with CLAI but it does not affect postoperative clinical scores. However, postoperative instability recurrence was more often observed in ankles with PTFL injuries, given that they frequently have poor-quality ATFL remnants and CFL injuries.

**Evidence level:**

Level III

## Introduction

Ankle sprains are the most common injuries that occur during sports and daily activities [1]. It has been reported that 10 to -30% of ankle sprains develop chronic lateral ankle instability (CLAI), which eventually results in osteoarthritis (OA) [2]. Therefore, CLAI should be treated appropriately to improve sports and daily activities and prevent OA progression. The lateral ankle ligaments consist of the anterior talofibular ligament (ATFL), calcaneofibular ligament (CFL), and posterior talofibular ligament (PTFL), and the ATFL is injured in 85%, CFL in 35%, and PTFL in 12% of all ankle sprains [3]. A recent anatomical study revealed that the ATFL is divided into two distinct fibers, superior and inferior fascicles, and the ATFL inferior fascicle (if) is connected to the CFL by arciform fibers, called the lateral fibulotalocalcaneal ligament (LFTCL) complex [4]. Even with isolated ATFL superior fascicle (sf) injury, dysfunction of the ATFLsf can induce notable deforming forces on other ligaments, which may result in the dysfunction of the LFTCL complex and PTFL. For patients with CLAI, lateral ligament repair such as the Broström procedure has been performed. Recently, arthroscopic lateral ankle repair has been widely performed with good clinical outcomes [5–7]. The arthroscopic procedure can repair the ATFLsf, which is an intra-articular ligament, but other unrepaired ligaments may affect long-term outcomes. In open procedures, whether to repair CFL in addition to ATFL repair is still controversial. Indeed, it has been reported that 30% of patients show OA changes in the ankle after the Broström procedure with isolated ATFL repair, although good clinical scores were obtained in the long-term follow-up [8]. Therefore, in addition to the arthroscopic ATFL repair, several procedures have been developed with the aim to repair CFL function. Vega et al. demonstrated arthroscopic all-inside ATFL and CFL repair for patients with a joint capsule opening, in which the CFL can be accessed through the intraarticular approach [9]. Other reports have shown good clinical outcomes of lateral ankle ligament repair in which CFL repair was performed when residual instability was observed immediately after arthroscopic ATFL repair [10, 11]. Despite many reports on the importance of ATFL and CFL in CLAI, few studies have investigated PTFL in CLAI, especially the clinical outcomes after surgery. It has been reported that the PTFL plays an important role in ankle stability and that severe injury induces posterolateral ankle instability [12]. A recent cadaveric study demonstrated that subsequent transection of the CFL and PTFL after ATFL resection worsened the lateral ankle instability, which suggests postoperative outcomes for CLAI with ATFL, CFL and PTFL dysfunction would be worse than those without PTFL [13]. What is known about PTFL injury in the CLAI is that the increased ligament thickness of the PTFL is observed as the morphological changes that subsequently occur in chronic ankle ligament injuries [14]. Although instability recurrence after arthroscopic ATFL repair has been reported, PTFL injuries have not been investigated. Therefore, the role of the PTFL injury in poor clinical outcomes remains unclear. We hypothesized that CLAI with PTFL injury might affect postoperative outcomes, even if the ATFL and CFL are repaired. The purpose of this study was to evaluate the magnetic resonance imaging (MRI) findings of the PTFL in patients with CLAI and determine whether the presence or absence of PTFL injury affected the postoperative outcomes of arthroscopic ATFL repair. This study will provide the impact of PTFL injury on CLAI outcomes in addition to our previous knowledge of CLAI treatment.

## Materials and methods

### Participants

Forty ankles of 35 patients who underwent arthroscopic ATFL repair for CLAI between March 2020 and December 2022 were retrospectively reviewed in this study. The patients comprised 13 males and 22 females, with a mean age of 33.5 ± 16.5 (range: 14–62) years. Patients who underwent preoperative thin-slice MRI and arthroscopic lateral ankle ligament repair were included. Patients with subfibular ossicles greater than 5 mm in size, painful os trigonum, osteochondral lesion of the talus or tibia, or systemic diseases, such as rheumatoid arthritis, were excluded.

Thirty ankles of 29 patients without CLAI were included as the control group to compare the MRI findings of the PTFL. These control patients comprised 14 males and 15 females, with a mean age of 27.3 ± 14.6 (range: 14–66) years. They did not complain of any instability of the ankle joint and had an MRI of the ankle joint taken at the same institution and with the same conditions as the CLAI group. The control patients had osteochondral lesions of the talus or tibia and loose bodies in the ankle joint. The absence of CLAI was confirmed using stress radiography and arthroscopic examinations. This study was approved by the local ethics committee of our university, and informed consent was obtained from all participants.

### Stress radiographs

Varus stress radiography was performed with a force of 50 N using a tension device (Imada, Toyohashi, Japan) preoperatively and 1 year postoperatively, and the talar tilt angle (TTA) was measured.

### MRI

**Fig. 1:**
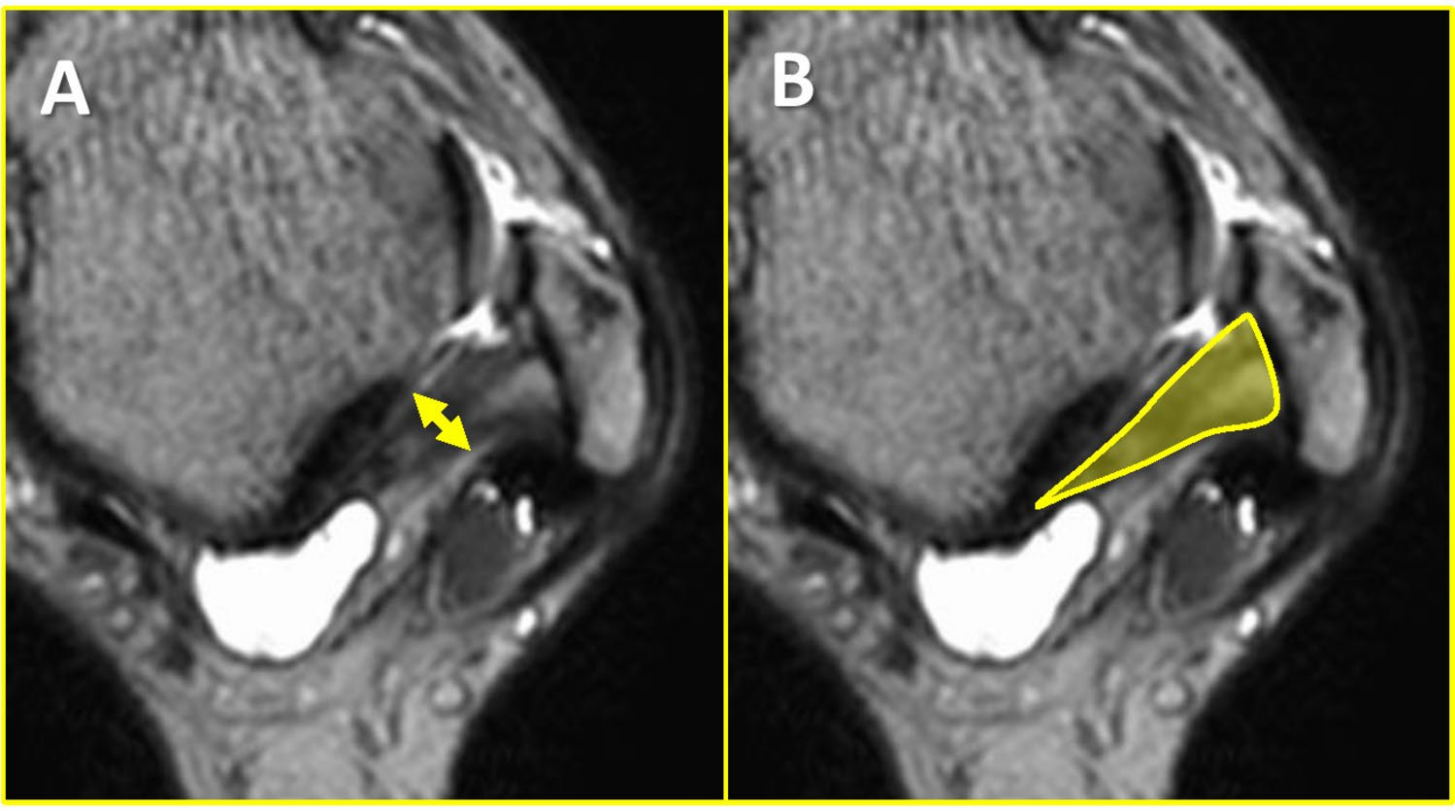
Evaluation of posterior talofibular ligament (PTFL) injury on magnetic resonance imaging (MRI). **A** Measurement of PTFL thickness (PTFLT) **B**. Measurement of the PTFL cross-sectional area (PTFLCSA)

MRI was performed using a 1.5 Tesla (T) MR unit (PHILIPS 1.5 T Achieva Release 3.2.3.4) with a dedicated foot/ankle coil. The following sequences were obtained: 3D T2-weighted images, TR, 1500 ms; TE, 75 ms, with a FOV of 150 mm, matrix of 240 × 240, and a slice thickness of 0.8 mm with a -0.4 mm gap. During the MRI scan, the ankle joint was held at 0-degree parallel to the long axis of the ATFL, and axial images were acquired. Axial images were used for the ATFLsf, ATFLif, and PTFL, according to a previous report [15]. For CFL evaluation, a slice parallel to the CFL was created on a workstation as an oblique CFL image. MRI findings of these ligaments showing absent, thin, stretched, irregular, or wavy, and thickened with or without an increase in intra-ligamentous signal intensity were diagnosed as ligament injury as previously described [15, 16]. On axial images, the PTFL thickness (PTFLT) and PTFL cross-sectional area (PTFLCSA) were evaluated according to a previous report [17]. The PTFLT was measured as the thickness between the anterior and posterior fibers of the PTFL (Fig. 1A), while the PTFLCSA was measured as the cross-sectional area of the PTFL (Fig. 1B).

### Clinical evaluation

Three scoring systems, including the Japanese Society for Surgery of the Foot (JSSF), Karlsson-Peterson (K-P) score, and Self-Administered Foot Evaluation Questionnaire (SAFE-Q), were used preoperatively and at the final follow-up to assess the clinical outcomes [18–20]. The SAFE-Q included the following five sub-scales: (1) pain and pain-related, including visual analogue scale, pain in the morning, at the end of the day, in sleeping, and in walking in several conditions in the past week; (2) physical functioning, including difficulty in going up- and downstairs, squatting, putting on socks, up- and downhill, and walking flat and uneven ground; (3) social functioning, including difficulty in going out, performing routine activities, enjoying leisure activities, and doing work and school activities; (4) shoe-related, inlcuding difficulty in putting high-fashion or formal shoes; and (5) general health and well-being, including mental state including anxiety, depression, and frustration [21, 22]. In addition, the history of ankle sprains after surgery was evaluated.

### Surgical procedure

Arthroscopic ATFL repair was performed according to a previous report [10, 11, 23]. Standard anterolateral (AL) and anteromedial (AM) portals were made, and a 30-degree oblique arthroscope was introduced. The ATFL remnants were divided into three groups according to the arthroscopic findings, as follows: (1) excellent, defined as arthroscopic observation of normal synovial tissue demonstrating a ligament with sharply defined margins; (2) moderate, defined as arthroscopic observation of fibrotic tissue or synovial tissue demonstrating a stretched hyperplastic or hypoplastic ligament; and (3) poor, defined as arthroscopic observation of a clear hypoplastic ligament with poorly defined margins [23]. An accessory AL portal (AAL) was created along the anterior border of the fibula as the working portal and was, viewed from the AL portal. Three knots of No.2 thread (Ultra-braid, Smith & Nephew, Memphis, TN, USA) or suture tape (Ultra-tape, Smith & Nephew) were placed at the ATFL remnant, and a drill hole was created at the ATFL footprint in the fibula. A knotless anchor (BIORAPTOR; Smith & Nephew) with No. 2 thread or suture tape was inserted and tightened with the ankle joint in a neutral position. Subsequently, varus stress and anterior drawer tests were performed manually after the procedure, and the TTA and anterior translation of the talus were measured using fluoroscopy. If the TTA and anterior translation remained after ATFL repair, CFL repair was performed [10, 11]. The AAL portal was extended approximately 15 mm toward the fibular tip, exposing the CFL. A soft anchor (Q-Fix mini; Smith & Nephew) was inserted into the CFL footprint under direct visualization. One end of the anchor suture was passed into the fibular side of the CFL and tied using the sliding knot technique. A varus stress test was performed after the CFL repair to confirm the absence of instability. Since it was confirmed that there were no ankles with the rupture on the talar side of the ATFL or the calcaneal side of the CFL by MRI findings in this series, ATFL and CFL were theoretically repaired in this procedure. Jogging was permitted at 6 weeks, and specific training for competitive sports was started at 8 weeks, with the aim of returning to competitive sports 12 weeks after surgery.

## Statistics analysis

Post hoc power analysis using G*Power for effect size was performed. For the calculation of post hoc analysis, we employed effect size 0.8 by Cohen’s interpretation guideline because the nature of discipline-specific effects is unknown in the previous study. For the primary aim of this study, ankles with and without PTFL injury were compared using the Mann- Whitney U test. To assess the rationale of the evaluation of the PTFL injury by PTFLT and PTFLCSA, the Mann- Whitney U test was also used to determine the significant differences in PTFLT and PTFLCSA between the control and CLAI groups. The JSSF scale, K-P score, SAFE-Q score, and TTA were compared pre- and postoperatively using paired *t*-test. Significant differences among the three groups of the ATFL remnant (Excellent, moderate, and poor groups) were analyzed using the Tukey–Kramer post hoc test. All statistical analyses were performed using SPSS for Windows, version 22.0 (IBM Corp., Armonk, NY, USA). Statistical significance was set at P < 0.05 for primary outcomes. Multivariable logistic regression with a forced entry method was performed to detect parameters influencing recurrent instability. Six parameters were selected as explanatory variables, including age, gender, BMI, TTA, MRI findings of PTFL, and ATFL remnant quality. The variables were categorized as negative or positive for abnormal findings of PTFL on MRI, and as excellent-moderate or poor for ATFL remnant quality. These analyses were performed using IBM SPSS Statistics for Windows, version 27.0 (IBM Corporation, Armonk, NY, USA).

## Results

**Fig. 2:**
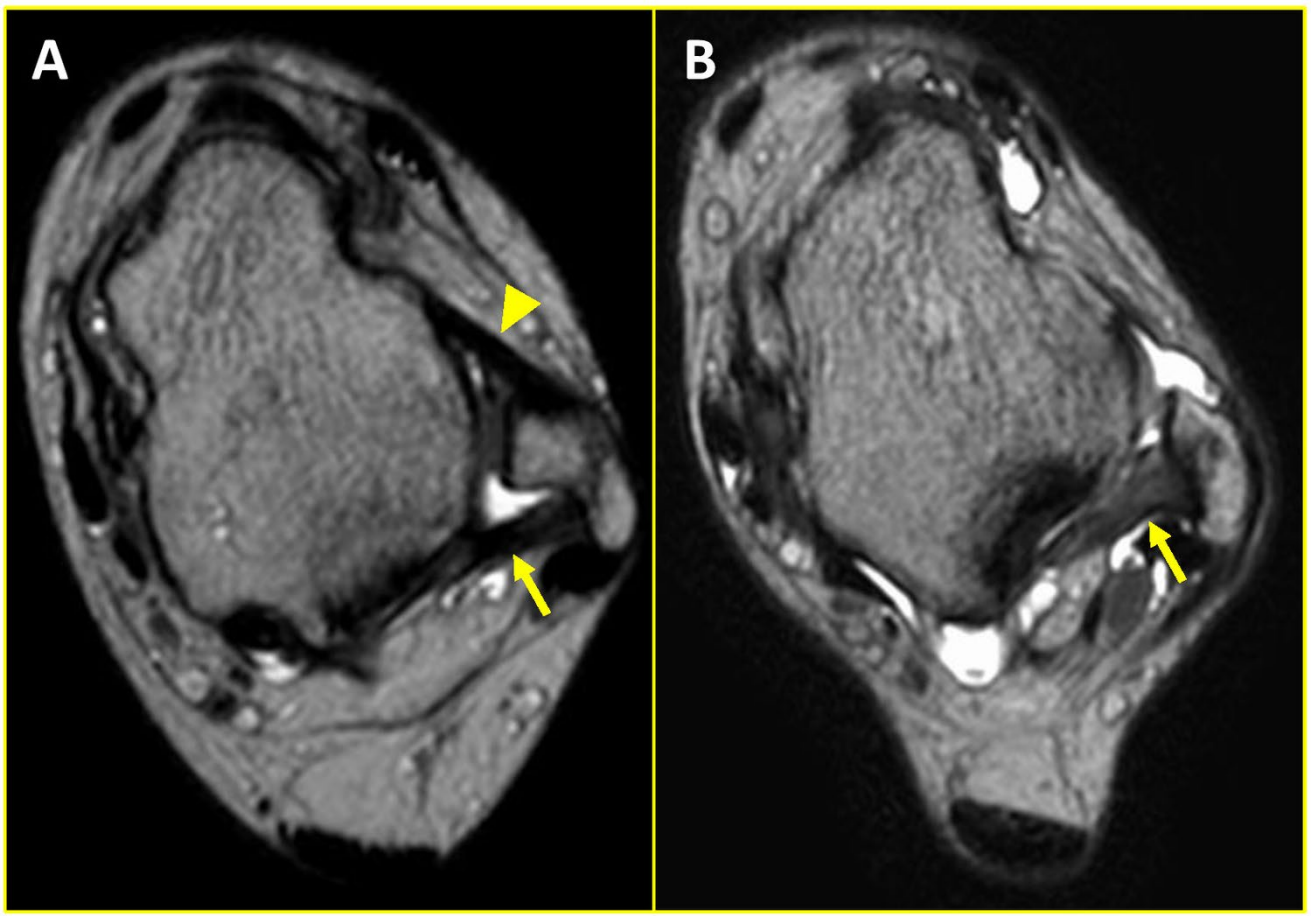
Comparison of the PTFL in the ankle with or without the chronic lateral ankle instability (CLAI). **A** Control group. **B** CLAI group. Arrows: PTFL, Arrowhead: Anterior talofibular ligament (ATFL)

There were no significant differences in mean age and BMI between the control and CLAI groups (Table 1). In the control group, the PTFL was observed as a straight, low-intensity fiber that ran from the medial aspect of the lateral malleolus of the fibula to the lateral tubercle of the posterior process of the talus (Fig. 2A). In contrast, the injured PTFL exhibited hypertrophic, irregular, or wavy fibers with increasing intra-ligamentous signal intensity (Fig. 2B). The PTFLT and PTFLCSA in the CLAI group were significantly larger than those in the control group (P < 0.01) (Table 1).

**Table 1:** Comparison of age, body mass index (BMI), PTFL thickness (PTFLT), and PTFL cross-sectional area (PTFLCSA) in the control and CLAI groups

	Control (n = 30)	CLAI (n = 40)	P value
Age (years)	27.3 ± 14.6 (14–66)	33.5 ± 16.5 (14–62)	0.231
BMI (Kg/mm^2^)	23.3 ± 3.8 (18.9–32.6)	23.6 ± 3.1 (17.2–30.2)	0.379
PTFLT (mm)	3.1 ± 0.3 (2.-3.6)	4.1 ± 0.9(2.3–6.1)	< 0.01
PTFLCSA (mm^2^)	56.8 ± 6.3 (47.0–71.0)	72.7 ± 21.8(43–110)	< 0.01

**Fig. 3:**
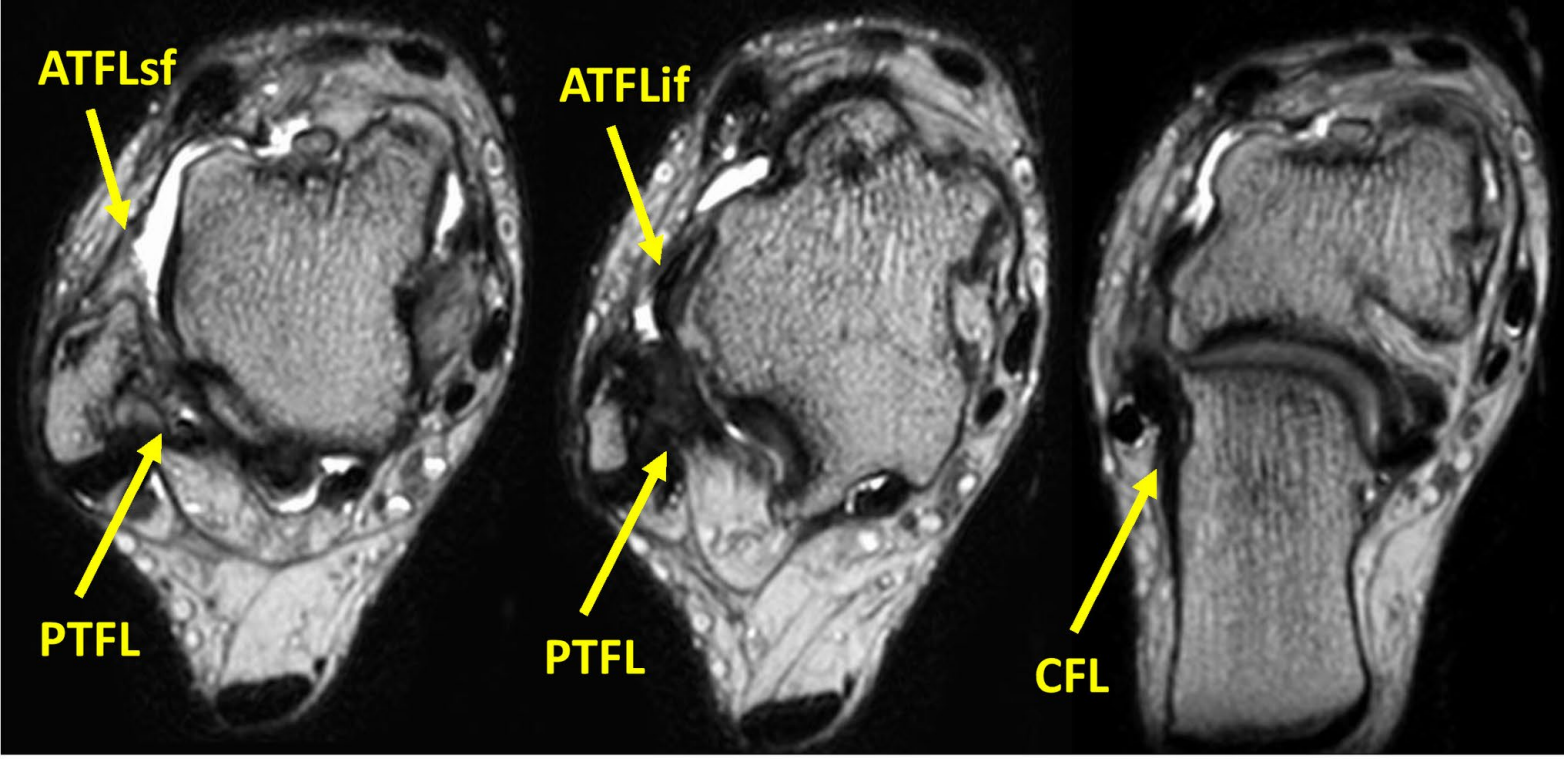
Representative images of the CLAI with ATFL superior and inferior fascicles, CFL, and PTFL

In the CLAI group, the JSSF scale and K-P score were significantly improved from the preoperative period to the final follow-up. Regarding the SAFE-Q, all subscales improved from preoperative to the final follow-up. The TTA was significantly improved from 12.1 ± 4.4 degrees to 4.7 ± 2.9 degrees (Table 2). Eighteen ankles required additional CFL repair, and 11 ankles exhibited recurrent instability. The mean postoperative TTA in the ankles with recurrence was 7.6 ± 3.7 (range: 3—17) degrees. On MRI, PTFL injury was observed in 28 ankles (70%). Eighteen ankles required additional CFL repair, all of which exhibited PTFL injury. When comparing the ankles with or without the PTFL injury, the PTFLT and PTFLCSA in the PTFL injury group were significantly larger than those in the non-injury group (P < 0.01). However, there were no significant differences in the clinical scores or postoperative TTA between the two groups. On MRI, ATFLif and CFL injuries were observed in 22 ankles (78.6%) in the PTFL injury group (Fig. 3). In contrast, ATFLif and CFL injuries were observed in one ankle (8.3%) and five (41.7%), respectively, in the non-injury group. Additional CFL repair was required in 18 ankles (64.3%) in the PTFL injury group. Four ankles were not performed additional CFL repair because instability was no longer present under varus and anterior stress tests after ATFL repair, even though preoperative MRI revealed CFL injury. On the other hand, ankles without PTFL injuries did not require additional CFL repair. Instability reoccurred in nine ankles (32.1%) in the PTFL injury group and two ankles (16.7%) in the non-injury group (Table 3).

**Table 2:** Comparison of the Japanese Society of Surgery of the Foot (JSSF), Karlsson-Peterson score, Self-Administered Foot Evaluation Questionnaire (SAFE-Q), and talar tilt angle (TTA) preoperatively and postoperative in patients with CLAI (n = 40)

	Preoperative	Postoperative
JSSF scale (points)	69.9 ± 4.9 (49–74)	96.4 ± 5.9 (82–100)**
K-P score	66.1 ± 5.3 (58–70)	95.0 ± 6.7 (80–100)**
SAFE-Q pain (points)	70.3 ± 11.0 (47.7–88.3)	95.7 ± 5.6 (85–100)**
SAFE-Q physical (points)	80.1 ± 11.1 (59.1–100)	97.1 ± 5.1 (85–100)**
SAFE-Q social (points)	79.0 ± 14.8 (29.2–100)	98.1 ± 3.5 (90–100)**
SAFE-Q shoe (points)	79.4 ± 15.7 (33.3–100)	97.7 ± 5.2 (75–100)**
SAFE-Q general (points)	73.2 ± 17.5 (25–100)	96.9 ± 5.0 (80–100)**
TTA (°)	12.1 ± 4.4 (4–20)	4.7 ± 2.9 (0–17)**

**P < 0.01

**Table 3:** Comparison of pre-and postoperative parameters in the ankles with and without PTFL injury. The P-value for recurrent instability could not be calculated due to the study not being powered for this analysis

	PTFL injury (−) (n = 12)	PTFL injury ( +) (n = 28)	P value
Age (years)	30.6 ± 16.3 (16–53)	34.8 ± 16.8 (14–62)	0.516
Male: Female	6: 6	8: 12	
BMI (Kg/mm^2^)	23.9 ± 3.1 (19.2–27.8)	23.5 ± 3.1 (17.2–30.2)	0.638
Follow up duration (months)	13.6 ± 3.7 (12–24)	15.9 ± 9.2 (12–55)	0.562
Preoperative JSSF scale (points)	71.7 ± 2.5 (64–74)	69.2 ± 5.5 (49–74)	0.25
Postoperative JSSF scale (points)	96.2 ± 7.2 (82–100)	96.5 ± 5.4 (58–70)	0.905
Preoperative K-P score (points)	67.2 ± 5.2 (58–70)	65.6 ± 5.4 (58–70)	0.39
Postoperative K-P score (points)	95.6 ± 7.7 (80–100)	94.8 ± 6.4 (80–100)	0.535
Preoperative SAFE-Q pain (points)	67.3 ± 11.3 (52.0–86.6)	71.6 ± 10.8 (47.7–88.3)	0.126
Preoperative SAFE-Q physical (points)	79.6 ± 8.8 (68.2–90.9)	80.3 ± 12.1 (65.0–97.7)	0.849
Preoperative SAFE-Q social (points)	73.3 ± 20.6 (29.2–100)	81.5 ± 10.9 (62.5–100)	0.294
Preoperative SAFE-Q shoe (points)	81.3 ± 14.3 (66.7–100)	78.6 ± 16.4 (33.3–100)	0.87
Preoperative SAFE-Q general (points)	70.8 ± 17.7 (40–95)	74.2 ± 17.6 (40–100)	0.617
Postoperative SAFE-Q pain (points)	96.5 ± 4.5 (90–100)	95.3 ± 6.0 (85–100)	0.728
Postoperative SAFE-Q physical (points)	97.5 ± 5.8 (85–100)	96.9 ± 4.9 (85–100)	0.384
Postoperative SAFE-Q social (points)	97.9 ± 4.0 (90–100)	98.2 ± 3.4 (90–100)	0.952
Postoperative SAFE-Q shoe (points)	98.4 ± 3.0 (91–100)	97.3 ± 5.9 (75–100)	0.93
Postoperative SAFE-Q general (points)	98.3 ± 3.3 (90–100)	96.3 ± 5.5 (80–100)	0.384
Preoperative TTA (°)	10.5 ± 3.3 (6–16)	12.7 ± 4.6 (4–24)	0.126
Postoperative TTA (°)	4.1 ± 1.7 (2–7)	5.0 ± 3.2 (0–17)	0.542
Recurrence of instability	2 (16.7%)	9 (32.1%)	
PTFLT (mm)	3.3 ± 0.6 (2.8–4.4)	4.4 ± 0.9 (2.3–6.1)	< 0.01
PTFLCSA (mm^2^)	58.5 ± 9.9 (43.3–75.0)	81.2 ± 18.0 (47.0–110.2)	< 0.01
CFL repair	0	18 (64.3%)	
ATFLif injury	1 (8.3%)	22 (78.6%)	
CFL injury	5 (41.7%)	22 (78.6%)	

The CLAI group was divided into three groups (excellent, moderate, and poor) according to the arthroscopic findings of the ATFLsf remnant, and various parameters including clinical outcome and PTFL findings on the MRI. Excellent remnant in seven ankles (17.5%), moderate in 19 ankles (47.5%), and poor in 14 ankles (35%) were observed. PTFL injuries were observed in two ankles (28.6%) in the excellent ATFL remnant group, 13 ankles (68.4%) in the moderate group, and 13 ankles (92.9%) in the poor group. ATFLif injuries in moderate and poor ATFLsf remnants were observed in 10 ankles (52.6%) and 13 (92.9%) ankles, respectively. No ATFLif injury was observed in the excellent ATFLsf remnants. CFL injuries in the excellent, moderate, and poor ATFLsf remnants were observed in 3 (42.9%), 12 (63.2%), and 12 ankles (85.7%) ankles, respectively. Postoperative scores were not significantly different with the numbers available. However, postoperative TTA and PTFLT were larger in the poor remnant group than in the excellent remnant group, and PTFLCSA in the poor remnant group was larger than in other groups (Table 4). Multivariable logistic regression analysis indicated that age (β = 0.893; p = 0.028), BMI (β = 1.859; p = 0.021), and poor quality of ATFL remnant (β = 20.141; p = 0.036) were factors significantly associated with recurrent instability (Table 5).

**Table 4:** Comparison of pre-and postoperative parameters in the excellent, moderate, and poor ATFL remnants

	Excellent (n = 7)	Moderate (n = 19)	Poor (n = 14)	significant differences
Age (years)	23.7 ± 12.1 (17–50)	36.7 ± 17.0 (14–62)	34.0 ± 16.9 (14–61)	ns
Male: Female	3: 4	7:12	4:10	
BMI (Kg/mm^2^)	22.3 ± 2.4 (19.2–25.3)	24.9 ± 2.6 (19.5–30.2)	22.6 ± 3.3 (17.2–28.2)	Moderate-poor; P < 0.01, excellent-moderate; P < 0.05
Follow up duration (months)	13.4 ± 2.5 (12–18)	14.9 ± 5.8 (12–33)	16.6 ± 11.7 (12–55)	ns
Preoperative JSSF scale (points)	72.3 ± 1.4 (70–74)	69.5 ± 6.0 (49–74)	69.3 ± 4.3 (64–74)	ns
Postoperative JSSF scale (points)	97.4 ± 6.8 (82–100)	96.0 ± 6.4 (82–100)	96.4 ± 5.0 (90–100)	ns
Preoperative K-P score (points)	72.3 ± 1.4 (70–74)	66.3 ± 5.4 (558–70)	64.6 ± 5.4 (58–70)	ns
Postoperative K-P score (points)	97.4 ± 7.9 (80–100)	94.3 ± 7.0 (80–100)	95.6 ± 6.1 (80–100)	ns
Preoperative SAFE-Q pain (points)	72.6 ± 12.1 (52–86.6)	67.8 ± 11.1 (47.7–88.3)	72.5 ± 10.3 (53–86.3)	ns
Preoperative SAFE-Q physical (points)	81.1 ± 8.6 (68.2–90.9)	80.8 ± 12.0 (59.1–100)	78.6 ± 11.5 (59.1–97.7)	ns
Preoperative SAFE-Q social (points)	75.0 ± 22.4 (29.2–100)	79.8 ± 13.8 (45.8–100)	80.0 ± 12.0 (62.5–100)	ns
Preoperative SAFE-Q shoe (points)	83.3 ± 13.6 (66.7–100)	77.6 ± 16.7 (33.3–100)	79.8 ± 15.9 (41.7–100)	ns
Preoperative SAFE-Q general (points)	77.9 ± 13.8 (55–90)	67.4 ± 20.4 (25–100)	78.7 ± 12.4 (65–100)	Moderate-poor; P < 0.05
Postoperative SAFE-Q pain (points)	97.3 ± 3.9 (90–100)	94.9 ± 5.9 (85–100)	95.9 ± 6.0 (85–100)	ns
Postoperative SAFE-Q physical (points)	97.9 ± 5.7 (85–100)	96.0 ± 5.5 (85–100)	98.1 ± 4.3 (85–100)	ns
Postoperative SAFE-Q social (points)	98.6 ± 3.8 (90–100)	97.6 ± 3.9 (90–100)	98.6 ± 3.1 (90–100)	ns
Postoperative SAFE-Q shoe (points)	97.3 ± 3.6 (91–100)	99.0 ± 2.7 (90–100)	96.1 ± 7.8 (75–100)	ns
Postoperative SAFE-Q general (points)	99.3 ± 1.9 (95–100)	96.6 ± 4.7 (85–100)	96.1 ± 6.3 (80–100)	ns
Preoperative TTA (°)	9.9 ± 4.6 (4–16)	11.2 ± 4.0 (7–24)	14.3 ± 4.0 (7–20)	Excellent-poor; P < 0.01, moderate-poor; P < 0.01
Postoperative TTA (°)	3.3 ± 1.6 (0–5)	4.5 ± 3.6 (2–17)	5.8 ± 1.7 (3.0–20)	Excellent-poor; P < 0.05
Recurrence of instability	0	3 (15.8%)	7 (50%)	
PTFL injury	2 (28.6%)	13 (68.4%)	13 (92.9%)	
PTFLT (mm)	3.4 ± 0.5 (3.1–4.4)	4.1 ± 0.9 (3.0–5.3)	4.4 ± 1.0 (2.3–6.1)	Excellent-poor; P < 0.01
PTFLCSA (mm^2^)	59.9 ± 9.4 (47–68)	77.8 ± 22.2 (43.0–110.0)	77.0 ± 15.0 (49.3–102.0)	Excellent-moderate: P < 0.05, excellent-poor; P < 0.05
ATFLif injury	0	10 (52.6%)	13 (92.9%)	
CFL injury	3 (42.9%)	12 (63.2%)	12 (85.7%)

**Table 5:** Multivariable logistic regression analysis for factors that influence recurrent instability

	Recurrent instability			
Explanatory variables	β	95% CI		P value
		Lower	Upper	
Age	0.893	0.807	0.988	0.028
BMI	1.859	1.1	3.141	0.021
ATFL remnant quality	20.141	1.255	331.108	0.036

Explanatory variables were age, gender, BMI, TTA, MRI findings of PTFL, and ATFL remnant quality. Β; standardized partial regression coefficient, *CI*; confidence interval

## Discussion

Our results revealed that 70% of the ankles with CLAI had PTFL injuries. However, there were no significant differences in clinical scores after arthroscopic ATFL repair between ankles with and without PTFL injury. PTFL injury was significantly associated with ATFLif and CFL injuries and poor ATFLsf remnants. Ankles with PTFL injuries had a higher rate of instability recurrence than those without PTFL injuries, even when additional CFL repair combined with arthroscopic ATFL repair was performed. The clinical implication of this study is that the lateral ligament complex with PTFL injury may affect instability recurrence after lateral ankle ligament repair, although it does not influence the postoperative clinical score.

The PTFL is the strongest ligament among the lateral ankle ligaments and is rarely injured. However, it has been reported that morphological changes in the PTFL are observed in CLAI [14, 17]. In CLAI, the dysfunction of other ligaments may provide mechanical stress to the PTFL, resulting in morphological changes. Recently, arthroscopic ATFL repair has been widely performed; therefore, we investigated the effects of PTFL injury on the clinical outcomes of ATFL and CFL repairs. In our study, there were no significant differences in clinical scores and postoperative TTA. Ozeki et al. reported that the PTFL is an important stabilizer especially when the ankle is in the dorsiflexed position [24]. Colville et al. demonstrated that the strain of the PTFL increases in dorsiflexion and plantarflexion, but is only minimally affected by ankle inversion [25]. Moreover, Pasmussen et al. reported that the PTFL plays a supplementary role in lateral ankle stability, particularly when the ATFL and CFL are intact [26]. In our series, CFL repair was performed when residual instability was observed after arthroscopic ATFL repair, indicating that ATFL and CFL deficiencies were eliminated immediately after surgery. In ankles with a well-repaired ATFL and CFL, injury of the PTFL, which plays a supplementary role in stability, may not affect clinical outcomes.

Anatomical features of the fiber connections in the lateral ankle ligaments may contribute to the lack of poor clinical outcomes in the ankles with PTFL injury. In a cadaveric study, Dalmau-Pastor et al. investigated the connections between the lateral ankle ligaments. They found connections between the ATFLif and PTFL in all ankles, and 42.5% of ankles showed connections between the ATFLsf and PTFL. In addition, connections between the CFL and PTFL were found in all ankles [27]. Furthermore, in an MRI study, connecting fibers from the ATFL to the PTFL were observed in 63.3% of ankles, and connections between the CFL and PTFL were identified in 70% of the ankles [28]. These reports revealed that most ankles have connections between the PTFL and CFL, indicating that CFL repair can provide tension to the PTFL. Some ankles have connections between the ATFL and PTFL, and ATFL repair can provide tension to the PTFL. Therefore, there are no significant differences in the clinical scores after ATFL repair or ATFL and CFL repairs.

In our results, ankles with PTFL injuries had a higher number of instability recurrences than those without PTFL injuries, although there was no significant difference in postoperative clinical scores. The recurrence of postoperative instability remains a major concern in arthroscopic lateral ankle ligament repair, and poor quality of the ATFL remnant often results in significantly higher recurrent instability owing to the low strength of the scar tissue of the remnant [10, 11, 29]. In the present study, PTFL injuries were associated with poor ATFL remnants. Because the ATFLif is connected to the CFL by arciform fibers, our study also showed that not only CFL injuries but also ATFLif injuries were highly associated with PTFL injuries [4]. Previous studies have shown that the quality of ATFL remnants in ankles requiring ATFL and CFL repair is poorer than those requiring ATFL repair [15]. The high incidence of recurrent postoperative instability in ankle joints with PTFL injuries may be owing to the poor quality of the ATFL remnant rather than the PTFL injury, which was revealed by our multivariable logistic regression analysis. For the ankles with PTFL injury, augmentation using artificial ligament in addition to the ATFL and CFL repair or reconstruction should be considered because the remnant of the ATFL is often poor.

This study had several limitations. First, PTFL injury was evaluated using only MRI findings, without confirmation by direct visualization or arthroscopic exploration. Although MRI facilitates the analysis of PTFL disorders, it is difficult to distinguish whether the anterior or posterior fibers of PTFL are injured [30]. In addition, it has been reported that partial tear and an asymmetrical thickening of the PTFL can occur anywhere [31]. Therefore, the cross-sectional area of the PTFL, including the anterior and posterior fibers, was measured according to a previous report [17]. Moreover, a 0.8-mm thickness slice of MRI was used to, depict the entire length of the ligament [15]. Because there were no reports of PTFLT and PTFLCSA assessment on thin slice thickness MRI, we validated PTFL injury using the ankles without CLAI as controls, and its results indicated that this assessment method was valid for evaluating PTFL injury. However, the relationship between the values of PTFLT and PTFLCSA and the degree of the instability is unclear. Further analyses of the MRI findings of the PTFL and detailed instability assessments are needed to clarify this relationship. Second, ankle kinematics with no ATFL and CFL dysfunction and only PTFL dysfunction are unknown. Theoretically, in our procedure, there should be no dysfunction in either the ATFL or CFL immediately after surgery. Cadaveric studies or long-term follow-up using MRI exploring the effects of PTFL injury after ATFL and CFL repair are desirable. Finally, the sample size was small, and we excluded ankles with os trigonum which is highly associated with CLAI because os trigonum may affect the PTFLT and PTFLCSA [32]. The effect size for the comparison of the control (n = 30) and CLAI group (n = 40) was large (d = 0.9). However, there was no adequate power for comparison of postoperative outcomes with and without PTFL injury (d = 0.61), even though the primary aim of this study is to compare these two groups. In addition, the comparison of the three groups by ATFL remnant quality is underpowered to be statistically significant. The limited sample size and lack of statistically significant power necessitate framing these comparisons as exploratory in nature. Further studies with large populations are needed to explore the effect of the os trigonum with PTFL injury on clinical outcomes.

## Conclusion

This study revealed that PTFL injury is highly associated with CLAI; however, there were no significant differences in postoperative clinical scores, including TTA, between ankles with and without PTFL injury. However, recurrence of postoperative instability was more frequently observed in ankles with PTFL injuries. As ankles with PTFL injuries frequently have poor-quality ATFL remnants and CFL injuries, it is important to consider the surgical strategy to prevent the recurrence of instability after surgery.

## Data Availability

The datasets used and/or analyzed during the current study are available from the corresponding author on reasonable request.
